# Lymphatic filariasis remained silent until presentation with huge abdominal mass mimicking malignancy: a case report

**DOI:** 10.1128/asmcr.00073-25

**Published:** 2025-09-08

**Authors:** S. Kafle, B. Panthi

**Affiliations:** 1 Department of Pathology, Tribhuvan University Teaching Hospital (TUTH)/Maharajgunj Medical Campus (MMC) 92959, Kathmandu, Nepal; Rush University Medical Center, Chicago, Illinois, USA

**Keywords:** mesenteric cystic mass, lymphatic filariasis, mimicking malignancy, Nepal, diethylcarbamazine

## Abstract

**Background:**

Lymphatic filariasis (LF) is an endemic infection seen in the tropical and subtropical regions of the world, including Nepal. It is a mosquito-borne neglected tropical disease and is caused by a nematode parasite.

**Case Summary:**

We reported an unusual case of LF in a 50-year-old male who presented to a tertiary hospital in Kathmandu with a complaint of a palpable abdominal mass mimicking malignancy. A CT scan revealed a large mesenteric cystic mass measuring approximately 23 × 15 cm. These findings initially directed the clinicians toward a diagnosis of malignancy, leading to blood tests for cancer markers, for example, carcinoembryonic antigen, which showed slight elevation supportive of this suspicion. Given the cystic nature of the mass and intestinal obstruction, surgical intervention was performed to place a pigtail catheter for drainage, and a fluid sample was sent for cytopathological evaluation for malignancy as well as biochemical and microbiological evaluation. Initially, biochemical and microbiological findings were reported as normal. Cytopathological study on stained slides (Giemsa and Papanicolaou) revealed multiple threadlike microfilaria larvae, the morphology of which resembled *Wuchereria bancrofti*, confirming the diagnosis of LF. This was further supported by the microbiological studies confirming the presence of sheathed microfilaria larvae in the cyst fluid. The patient’s condition significantly improved after initiation of diethylcarbamazine. No further surgical intervention was required.

**Conclusion:**

LF may remain asymptomatic for decades. This case highlights that LF may present with an abdominal mass mimicking malignancy.

## INTRODUCTION

Lymphatic filariasis (LF) is a neglected tropical disease transmitted by mosquitoes. It is caused by various species of nematode parasite, common of them is *Wuchereria bancrofti,* which accounts for 90% of the cases. Some other parasites are *Brugia malayi* or *Brugia timori* (1). Mosquitoes acquire microfilariae when they feed on an infected host. The microfilariae then develop into infective larvae that are transmitted to humans during subsequent bites. It resides in the lymphatic system and can survive for 6–8 years, and releases millions of microfilariae into the bloodstream (2). The clinical features of LF are mainly related directly to the occlusion of lymphatic vessels, causing lymphangiectasia (3). It can be subclinical or cause various symptoms like hydrocele, lymphedema, elephantiasis, and acute episodes of inflammation in the lymph nodes (4). Rarely, it can present as a mesenteric cyst and may be associated with lymphatic malformations, trauma, or infections and may be asymptomatic or present with intestinal obstruction mimicking malignancy (5–7).

In Nepal, currently, 63 out of the 77 districts are endemic to filariasis, with the Tarai and subtropical regions being the most affected, which have favorable mosquito breeding conditions. The government of Nepal has launched the National Filariasis Elimination Program in 2003, aiming to eliminate LF as a public health issue by 2020. The goal was later extended to 2030 to align with the global targets set by the World Health Organization (WHO). The program targets mass drug administration (MDA) and morbidity management and disability prevention related to filariasis. It had already conducted the MDA program, administering a combination of diethylcarbamazine (DEC) and albendazole to at-risk populations once a year for six years. Additionally, it has introduced new programs using a triple-drug regimen of ivermectin, DEC, and albendazole in select districts (8). Nepal is still suffering from the curable and preventable disease—filariasis.

Here, we report an unusual presentation of LF as a mesenteric cystic lesion, whose diagnosis was confirmed by the demonstration of the microfilariae larva in the cytopathological analysis of cyst fluid.

This case report has been reported in line with the SCARE criteria (9).

## CASE PRESENTATION

A 50-year-old male from a nearby hilly region presented to the emergency department of Tribhuvan University Teaching Hospital, a tertiary hospital in Kathmandu, with a complaint of an abdominal mass causing acute intestinal obstruction. He complained of severe abdominal pain, vomiting, and constipation for just 2 days. He did not have fever, chills, or weight loss. There was no history of past medical illness requiring hospital admission or any history of abdominal trauma or surgeries. He also denied any shortness of breath, chest pain, or dysuria. Vital signs were stable.

**Fig 1:**
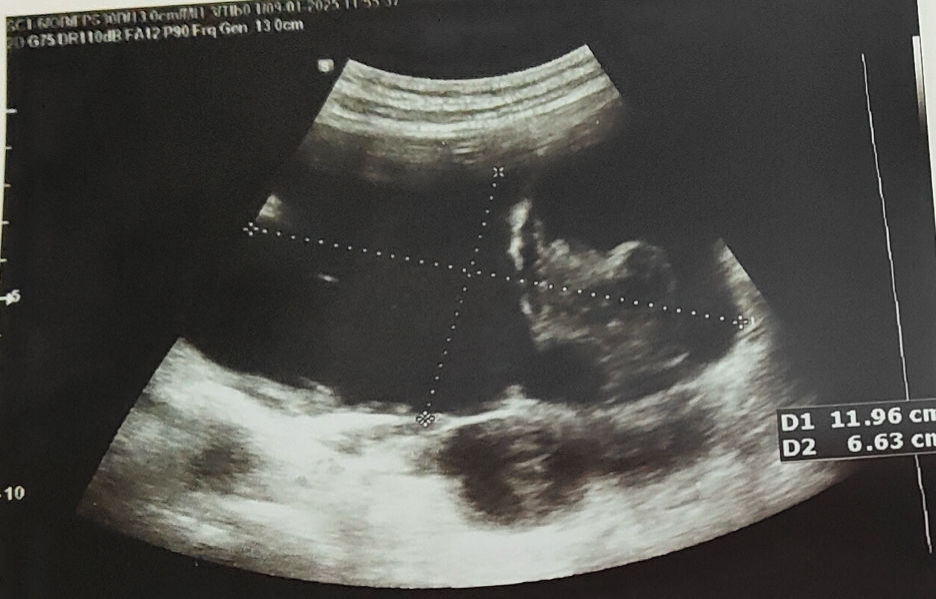
Ultrasound of the abdomen showed a mass of size 11.9 × 6.6 cm with multiloculated cyst having septations.

**Fig 2:**
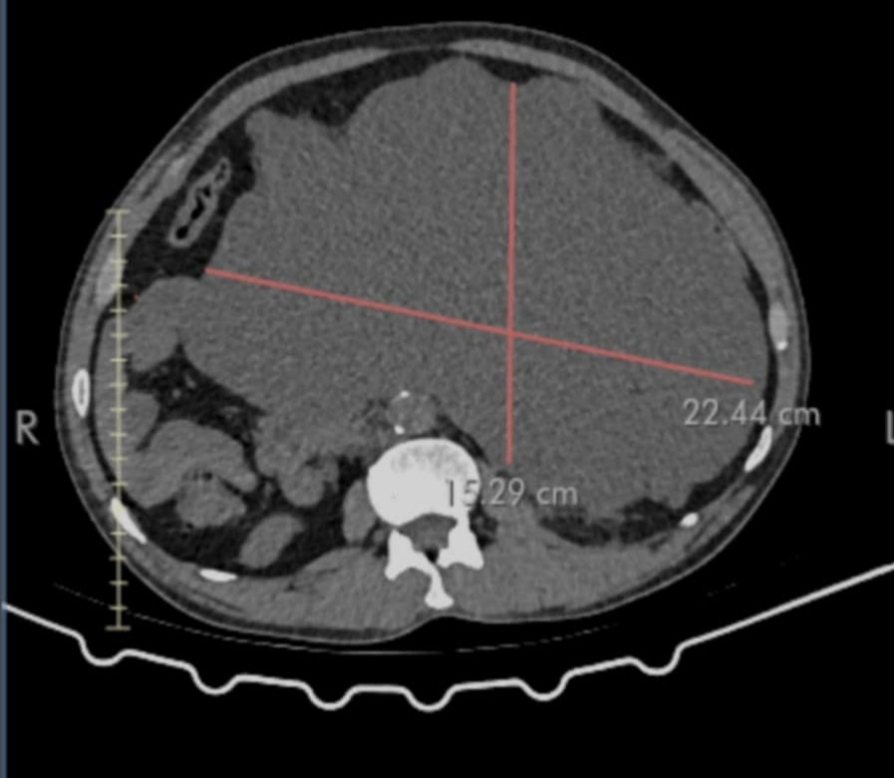
A computed tomography scan of the abdomen and pelvis revealed a larger, heterogeneous well-defined mass (22.44 × 15.29 cm) in the mesenteric region.

Physical examination revealed a distended abdomen with mild upper abdominal tenderness, mild guarding, and reduced bowel sounds. Murphy’s sign was negative, and there was no rebound tenderness or hepatosplenomegaly. Routine blood investigations were within normal limits. An X-ray of the abdomen showed dilated loops of the small bowel. Ultrasound of the abdomen showed an approximately 11.9 × 6.6 cm-sized multiloculated cystic lesion with internal septations in the retroperitoneum extending to the peritoneal cavity (Fig. 1). Computed tomography scan of abdomen and pelvis revealed a larger, heterogeneous, well-defined mass (22.44 × 15.29 cm) in the mesenteric region with suspicion of malignancy (Fig. 2).

A diagnosis of acute partial small bowel obstruction secondary to a mesenteric mass was made, and the patient was admitted to the surgical ward. The patient was initially managed conservatively with nil per oral, nasogastric tube insertion, intravenous fluids, antiemetics, and analgesics. Further laboratory workup revealed normal amylase, lipase, CA-19.9, and triglyceride levels, shown in Table 1. Carcinoembryonic antigen (CEA) was 7.84, which was slightly increased, supporting the suspicion of malignancy.

**TABLE 1:** Laboratory values of different tests (with institutional reference range)

Test	Result	Unit	Reference range
CEA	7.84	ng/mL	<3.0 ng/mL
CA19.9	<2	Unit/mL	<37 unit/mL
Amylase	39	Unit/L	25–125 Unit/L
Lipase	42	Unit/L	0–160 Unit/L
Triacylglycerol	0.6	Mmol/L	0.5–1.8 mmol/L

**Fig 3:**
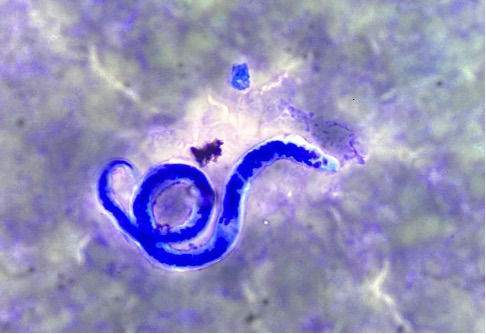
Microfilaria (larval stage of filarial parasite) in a Giemsa-stained cytopathological smear under oil immersion microscopic view (1000×).

Peripheral blood smear revealed normal findings. Due to the persistent obstruction, an ultrasound-guided 12 F locking pigtail was introduced into the mesenteric cyst, and the cyst fluid was sent for biochemical, microbiological, and cytopathological studies. Initially, biochemical and microbiological findings were normal. However, the cytopathological study of fluid stained with Giemsa and Papanicolaou revealed multiple threadlike microfilaria larva, the morphology of which resembled *Wuchereria bancrofti*, confirming the diagnosis of LF (Fig. 3).

Microbiological analysis was consistent with our diagnosis stated as “microfilaria identified- morphological consistent with *Wuchereria bancrofti.*”

Post-pigtail insertion, the patient developed features of acute hemorrhagic shock, for which he was shifted to intensive care and managed meticulously with IV fluids, blood transfusion, analgesics, antibiotics for possible peritonitis, and careful monitoring. Albendazole 400 mg single dose along with DEC 300 mg per oral per day after meal was given for 12 days. The patient showed significant improvement after the initiation of DEC therapy. His abdominal pain subsided, bowel sounds returned, and he was discharged from the hospital in a stable condition on day 7. On follow-up after the 12th day of commencement of DEC, the patient showed a significant symptomatic response.

## DISCUSSION

The clinical features of LF are mainly due to the occlusion of lymphatic vessels, causing lymphangiectasia and *W. bancrofti,* which is common in Asia, mainly presents with lymphangitis, fever, elephantiasis, hydrocele, and chyluria (3, 10). Presenting as an acute abdominal condition is very rarely seen (11). Our case presented as a mesenteric cyst. Such cases are extremely rare and may present as an intra-abdominal mass (5, 7). Despite clinical signs suggesting an acute abdominal pathology, initial laboratory tests, including complete blood count with differential and serum amylase, were done as stated by Roufosse et al. (12) and were within normal limits, similar to the cases reported by Metha et al. (11). Imaging findings, including ultrasonography and contrast-enhanced CT scan, are very effective in identify the benign as well as malignant lesions (11, 13). However, despite extensive imaging evaluation, the diagnosis can only be made after pathological analysis of cyst fluid, which was also supported by the findings in our case (6, 11).

On cytopathology as well as histopathology, especially with hematoxylin and eosin stains (H&E stain) and Giemsa stain, the microfilariae are identified as thread-like organisms having terminal and subterminal nuclei in the tail region (14). Our case, *W. bancrofti* in Giemsa-stained slide, was with colorless sheath, short headspace, and the tail was anucleate and tapered to a point. The nuclear column was loose, and individual nuclei could be visualized throughout the column (Fig. 3). These features were typical and differentiated from similar microfilaria like *B. malayi*, *B. timori,* and *Onchocerca,* which have been illustrated in tabulated form in Table 2 (15).

**TABLE 2:** Differentiating features of different species of filaria

Species	Length (μm)	Key diagnostic morphologic features
*Wuchereria bancrofti*	244–296	Usually colorless sheath (Giemsa), anucleate tail, short headspace and relatively loose nuclear column
*Brugia malayi*	177–230	Usually hot-pink sheath (Giemsa), terminal and subterminal tail nuclei separated by large gaps and long headspace
*Brugia timori*	310 (avg)	Usually colorless sheath (Giemsa), terminal and subterminal tail nuclei separated by large gaps and long headspace
*Onchocerca volvulus*	304–315	Sheath never present, tail tapered and often flexed and anucleate

Albendazole in combination with DEC is effective at treating LF in endemic areas. It works by clearing microfilariae and sterilizing adult filarial worms and is also used as a preventive chemotherapy strategy for the elimination of LF in a MDA program (2). Alternative drugs like doxycycline (a filaricidal drug with anti-*Wolbachia* action) could result in a successful eradication of microfilaricidal burden. Most filarial parasites of humans such as *W. bancrofti*, *B. malayi,* and *Oncocerca* spp depend on endosymbiotic *Wolbachia* bacteria for growth, development, fertility, and survival, whereas the host nematode likely supplies amino acids needed for *Wolbachia*’s development. Doxycycline permanently sterilizes female worms and reduces the adult worm longevity, inducing potent macrofilaricidal activity by the killing of adult worms. These properties are superior to other anti-filarial drugs like DEC, ivermectin, and albendazole having limited activity against adults and predominantly acting against the microfilaria progeny of female worms (16).

Usually, drug therapy is sufficient enough to resolve the symptoms and reduce the size of the cyst. However, in cases with larger size or chemotherapy resistance and with persistent obstruction, surgical removal may be the ultimate management (5). In our case, although the cyst size was large, premedical pigtail drainage already reduced the size of the cyst, and no further surgical intervention was required after medical therapy.

LF may remain asymptomatic for decades, and it may present with an abdominal mass mimicking malignancy. This case report emphasized the significance of considering LF as a differential diagnosis in patients presenting with abdominal mass or symptoms of acute intestinal obstruction, particularly in endemic areas.

### Highlights

– Lymphatic filariasis (LF) is a mosquito-borne neglected tropical disease and is caused by a nematode parasite.
– Various symptoms include hydrocele, lymphedema, elephantiasis, and acute episodes of inflammation in the lymph nodes, and rarely mesenteric cysts.
– Mesenteric cysts can present as intra-abdominal masses mimicking malignancy.
– Albendazole in combination with diethylcarbamazine is effective at treating LF in endemic areas
– LF could be a differential diagnosis in acute abdominal mass with suspicion of malignancy.
